# Magic Performances – When Explained in Psychic Terms by University Students

**DOI:** 10.3389/fpsyg.2018.02129

**Published:** 2018-11-06

**Authors:** Lise Lesaffre, Gustav Kuhn, Ahmad Abu-Akel, Déborah Rochat, Christine Mohr

**Affiliations:** ^1^Institute of Psychology, Social and Political Sciences, University of Lausanne, Lausanne, Switzerland; ^2^Department of Psychology, Goldsmiths University of London, London, United Kingdom

**Keywords:** belief, causality, cognitive bias, event probability, magic

## Abstract

Paranormal beliefs (PBs), such as the belief in the soul, or in extrasensory perception, are common in the general population. While there is information regarding what these beliefs correlate with (e.g., cognitive biases, personality styles), there is little information regarding the causal direction between these beliefs and their correlates. To investigate the formation of beliefs, we use an experimental design, in which PBs and belief-associated cognitive biases are assessed before and after a central event: a magic performance (see also Mohr et al., 2018). In the current paper, we report a series of studies investigating the “paranormal potential” of magic performances (Study 1, *N* = 49; Study 2, *N* = 89; Study 3, *N* = 123). We investigated (i) which magic performances resulted in paranormal explanations, and (ii) whether PBs and a belief-associated cognitive bias (i.e., repetition avoidance) became enhanced after the performance. Repetition avoidance was assessed using a random number generation task. After the performance, participants rated to what extent the magic performance could be explained in psychic (paranormal), conjuring, or religious terms. We found that conjuring explanations were negatively associated with religious and psychic explanations, whereas religious and psychic explanations were positively associated. Enhanced repetition avoidance correlated with higher PBs ahead of the performance. We also observed a significant increase in psychic explanations and a drop in conjuring explanations when performances involved powerful psychic routines (e.g., the performer contacted the dead). While the experimentally induced enhancement of psychic explanations is promising, future studies should account for potential variables that might explain absent framing and before–after effects (e.g., emotion, attention). Such effects are essential to understand the formation and manipulation of belief.

## Introduction

Paranormal beliefs (PBs) and associated phenomena are of substantial interest to both the general population and the scientific community. Survey data indicate that PBs,^1^ such as beliefs in telepathy, witchcraft, and precognition, are common in the general population (Rice, 2003; Moore, 2005; Knittel and Schetsche, 2012). In the laboratory, adults often explicitly deny PBs, but implicitly acknowledge paranormal interpretations of an event (Nemeroff and Rozin, 2000; Subbotsky and Quinteros, 2002; Subbotsky, 2004a,b). Psychologists have long been interested in paranormal and/or magical (including psi) phenomena in children as well as adults (Rosengren et al., 2000; Subbotsky, 2001, 2004a, 2010; Wiseman et al., 2003; Wiseman and Watt, 2004, 2006; Lindeman and Aarnio, 2006; Losh and Nzekwe, 2011; Risen, 2016). Some studies focused on the existence of paranormal phenomena (Honorton and Harper, 1974; Bem and Honorton, 1994; Milton and Wiseman, 1999; Etzold, 2006; Moulton and Kosslyn, 2008), while others focused on cognitive thought processes that underpin PBs (Brugger et al., 1990; Lindeman et al., 2008; Fiske and Taylor, 2013). Yet, others focused on personality traits (e.g., intelligence, creativity, schizotypy traits, extraversion, and reasoning abilities) associated with PBs (for reviews, see e.g., French, 1992; Irwin, 1993).

There are a variety of definitions of what constitute PBs. Woolley (1997) defined PBs as reasonings that are “based on some sort of misconception about causality, or about natural laws more generally” (Woolley, 1997, p. 993), while Keinan (1994) regarded PBs as “any explanation of a behavior or experience that contradicts the laws of nature... [and] usually refers to powers, principles, or entities that lack empirical evidence or scientific foundation” (Keinan, 1994, p. 48). Yet, others define PBs as *ontological beliefs* (Alcock, 1995; Subbotsky, 2004a), for example, as “the interpreting of two closely occurring events as though one caused the other, without any concern for the causal link” (Alcock, 1995, p. 15). Such definitions imply that cognitive processes, strategies, or biases underlie PBs (e.g., reasoning). By inference, individuals who demonstrate PBs should be more likely to use any of these cognitive processes mentioned in these definitions. Indeed, research indicates that PBs are associated with biases such as clustering illusions (Gilovich et al., 1985), availability error (Tversky and Kahneman, 1973), confirmation bias (Wason, 1960), illusion of control (Langer, 1975; Irwin et al., 2013), and the blind spot bias (Pronin et al., 2002). Individuals high in PBs seem to consider only part of available information, overestimate the occurrence of improbable events, and tend to over-attribute causal links between events that occurred close in time and space (Pratkanis, 1992; Taylor et al., 1995; Willingham, 2008; Fiske and Taylor, 2013). Further research indicates that individuals high in PBs show relatively deficient deductive (Lawrence and Peters, 2004), logical (Tsakanikos, 2004), and conditional (Sellen et al., 2005) reasoning abilities. Finally, participants with elevated compared to low PBs tend to see meaning in random events and stimuli (Brugger et al., 1993; Blackmore and Moore, 1994; Riekki et al., 2013).

While the above studies are important and informative, they are nevertheless correlational. It is, therefore, not possible to establish the causal direction between the cognitive biases and the PBs (see also Mohr et al., 2018). In the published literature, researchers assume that PBs are present in early childhood (Subbotsky, 2004a,b) and have a trait-like characteristic (e.g., Wiseman et al., 2003; Lindeman and Aarnio, 2006). This assumption implies that PBs in adulthood reflect potential residues from childhood. However, some laboratory studies have shown that people’s willingness to endorse paranormal phenomena is malleable. For example, verbal suggestions facilitated people’s experience of seeing a spoon bending (Wiseman et al., 2003), individuals’ perception of a psychic’s^2^ abilities (Wiseman and Greening, 2005), and enhanced their impression of being observed in a supposedly “haunted” room (Bering et al., 2005). Even scientists have been tricked into accepting a magician’s psychic abilities (see, e.g., Benassi et al., 1980; Randi, 1983a,b).

Benassi et al. (1980) showed that highly educated adults (including scientists) could be fooled into attributing psychic powers to ordinary magic performances. In their study, a magician performed a range of magic tricks in the classroom. Crucially, the magician was introduced as either a conjuror (conjuror condition)^3^ or a psychic (psychic condition). After the performance, individuals in the psychic as compared to the conjuror condition provided higher psychic explanations. Yet, more than half of the participants in either condition considered psychic explanations, i.e., also in the conjuror condition. This experimental manipulation implies that contextual framing (psychic vs. conjuror condition) seems to influence how people interpret a magic performance; however, the authors did not assess PBs and PB-related cognitive biases before and after the performance. Thus, causal inferences cannot be drawn from this report. Nevertheless, the paradigm of Benassi et al. (1980) offers interesting avenues to develop studies targeting PB formation (see also Mohr et al., 2018).

Mohr et al. (2015) investigated whether exposure to a magic performance in the classroom was able to change an individual’s PBs. As in Benassi et al. (1980), about half of the sample was told that the performer was a psychic, and the remainder that the performer was a conjuror. Explicit and implicit measures of PBs were measured both before and after the performance, using the Revised Paranormal Belief Scale (RPBS, Tobacyk, 2004) and repetition avoidance, respectively.^4^ After the performance, participants responded as to whether they would explain the event in psychic, conjuring, or religious terms. The group that received the psychic framing (the performer is a psychic; psychic group) demonstrated stronger repetition avoidance compared to the group that received the conjuror framing (the performer is a conjuror; conjuror group). However, the authors reported no differences between sessions (i.e., before–after magic performance) in either participants’ explicit (RPBs scores) or implicit (repetition avoidance) correlates of PBs. Participants’ baseline belief scores correlated, however, with higher psychic explanation ratings, and they gave explanations in intuitive ways: the psychic group gave more psychic explanations and the conjuror group gave more conjuring explanations. Yet, overall, the psychic explanation ratings were relatively low, indicating few participants endorsed the demonstration as being genuinely paranormal. We, therefore, aimed to explore ways in which the demonstration could be made more believable, and, thus, elicit stronger PBs.

The current series of studies builds on these observations (Benassi et al., 1980; Mohr et al., 2015), further elaborating on the paranormal potential of magic performances (see also Mohr et al., 2018). Magicians use a wide range of deceptive methods (Kuhn et al., 2014) to create impossible events (Kuhn, 2019). For example, sleight of hand and gimmicked devices can be used to create the illusion that it is possible to read a person’s mind, or even to communicate with the dead. This knowledge was used to enhance the “paranormal” nature of the magic performance. Furthermore, the actual formulations of the framing texts used for the psychic and conjuring conditions were aligned (i.e., same length, sentence structure, and message) so that only key statements/words differed. In the second study, we further enhanced the “paranormal” nature of the magic performance. In the third study, we implemented the procedure of the second study, and added a manipulation check to ascertain that the framing instructions had been read and understood. Participants were always tested in one session in the classroom, through booklets on which the framing was given in a written format, and the key measures, namely, PBs and repetition avoidance, were assessed before and after the performance. In addition, after the performance, we asked participants to rate the extent to which the event could be explained in psychic, conjuring, or religious terms. In line with our reasoning (see also Mohr et al., 2018), we expected that the psychic group would consider more psychic explanations than the conjuror group, and would yield stronger increases in PBs and repetition avoidance than the conjuror group. We also expected psychic explanations to be higher in the second and third studies compared to the first study.

## Materials and Methods

### Participants

Undergraduate psychology students were the attendant participants for Study 1 (*N* = 53), Study 2 (*N* = 95), and Study 3 (*N* = 170). Subsequent to data cleaning (see Section “Data Analysis” for details), we retained 49 students for Study 1 (67.3% females, *M*_age_ = 22.37 years, *SD* = 5.69); 89 students for Study 2 (82% females, *M*_age_ = 20.49 years, *SD* = 5.31); and 123 students for Study 3 (78% females, *M*_age_ = 20.54 years, *SD* = 5.40). Analyses were performed on the data from the remaining 261 participants (77.39% females, *M*_age_ = 20.87 years, *SD =* 5.45).

All studies took place after a lecture in a psychology undergraduate course on research methods, given by the second author (GK) at Goldsmiths University in London. Students participated in exchange for course credits. The study protocol was approved by the Goldsmiths Ethics Committee and followed the ethical guidelines of the Declaration of Helsinki (World Medical Association, 2013). Each participant provided written informed consent prior to the experiment.

### Measures Common to All Studies

#### Paranormal Belief

We used the RPBS (Tobacyk, 2004) to measure PBs. This 26-item self-report questionnaire consists of seven subscales measuring Traditional Religious Belief, Psi, Witchcraft, Superstition, Spiritualism, Extraordinary Life Forms, and Precognition. The four traditional religious belief items can be summed into a traditional belief score and the remaining items into a non-traditional belief score (see also Mohr et al., 2015). Item examples include “Some psychics can accurately predict the future” (non-traditional beliefs), “It is possible to communicate with the dead” (non-traditional beliefs), and “There is a heaven and hell” (traditional belief). Items are formulated such that participants are asked to answer along a seven-point Likert scale ranging from 1 (strongly disagree) to 7 (strongly agree). Accounting for reverse coded items, the scores are summed so that higher scores reflect greater beliefs. We had no *a priori* prediction that the different non-traditional beliefs subscales would be differentially sensitive to our manipulation; thus, we did not account for these. We account, however, for the possibility that traditional belief (or practices) are more sensitive to cultural influences than non-traditional beliefs (MacDonald, 1995; Orenstein, 2002). Accordingly, we used, respectively, the sum scores for the traditional belief items (*N* = 4) and the non-traditional beliefs items (*N* = 22). Normative values can be found in Tobacyk (2004), and a recent psychometric evaluation in Drinkwater et al. (2017).

#### Explanation

In line with Mohr et al. (2015), participants rated on a seven-point Likert scale (1 for strongly disagree to 7 for strongly agree) whether the performance was accomplished through (1) paranormal, psychic, or supernatural powers (psychic explanation), (2) ordinary magic trickery (conjuring explanation), or (3) religious miracles (religious explanation). Detailed instructions are presented in Supplementary Material.

#### Mental Dice Task

In the Mental Dice Task (MDT; Brugger et al., 1990), participants received written and verbal instructions to imagine throwing a dice each time they heard a beep and to write down the number that they imagined being on top of the dice (66 trials). Computer-generated beeps were played 66 times at 1 s intervals, during which the participant wrote down the imagined number. We calculated the repetitions in the number sequence (i.e., 1-1, 2-2, and 3-3). If the number generation were entirely random, we would expect participants to produce on average 11 repetitions. Previous research has shown that participants avoid repetitions, and that this repetition avoidance is stronger for individuals scoring high on non-traditional beliefs (Brugger et al., 1990).

#### Magic Performances

##### Study 1

The magic performance was performed by a professional magician (henceforth, “performer”), who was a member of the Magic Circle.^5^ The performer presented a range of magic tricks that were presented using an occult gothic theme. This performance included conventional magic tricks using magic props (e.g., cards, gimmicked locks). The tricks were similar to those used by Benassi et al. (1980). Unlike Mohr et al. (2015)’s single magic trick, the performer did several different magic tricks and the entire performance lasted for approximately 20 min. The performance started with a card trick, in which the performer demonstrated his telepathic abilities by allegedly reading the spectator’s mind to discover a chosen card. This was repeated for three randomly selected individuals. In another trick, a member of the audience was asked to choose one of several keys, which turned out to be the only key to open a box. The performer also demonstrated his telekinetic powers by giving the illusion that he was capable of moving a key simply though the power of his mind.

##### Study 2

We kept the same overall procedure from Study 1, but aimed for a stronger performance. After Study 1, students reported that they were not very impressed by the performance, and did not consider the event to be of psychic nature. In Study 2, similar to Study 1, the magic performance was done by a professional magician, who was a member of the Magic Circle. This time, a different person did the magic performance due to special circumstances (see Section “Acknowledgments”). We intentionally avoided using special magic props, and ensured that the demonstration would emulate a psychic reading typically encountered in public spiritualist reading. We predicted that such a demonstration would make the psychic nature appear more genuine. Here, the performance was divided into two parts. In the first part, the performer guessed the color on a dice^6^ a volunteer has selected (the dice is turned such that the selected color is shown on top). The performer indicated that he would do so by “catching the aura” of the volunteer. The performer tried it with five volunteers. He was false (deliberately, in order to make it “more real”) two out of five times – a technique commonly used by magicians to enhance the effectiveness of the illusion. In the second part, the performer invited a confederate from the audience to join him. From the audience’s perspective, the confederate was a randomly selected volunteer, who had been selected through a ballot. However, this ballot was rigged, which ensured we selected our confederate as the volunteer. The audience was not aware that this person was a confederate. This female confederate was asked to think about one of her deceased close family members, in order to get in touch with him/her. The performer, after “having felt” a presence, started to “guess” details about the person. He first suggested that the presence was her father, deceased 10 years ago. He reported more and more correct details about his life. These details were almost spot on (i.e., he guessed that his name was Zack, but it was actually Jack). The confederate became (i.e., acted) increasingly emotional. The performer finished his magic performance by telling the young woman that her father loves her, that he was very proud of her, and that he will always look after her.

##### Study 3

For Study 3, the same performer, as in Study 2, did the magic performance. The same magic performance was used, except for some changed details about the revelations (i.e., the deceased relative was the grandmother). The results from Study 2 revealed that the performance was very convincing, and we, therefore, implemented the same overall procedure.

### General Procedure

**FIGURE 1:**
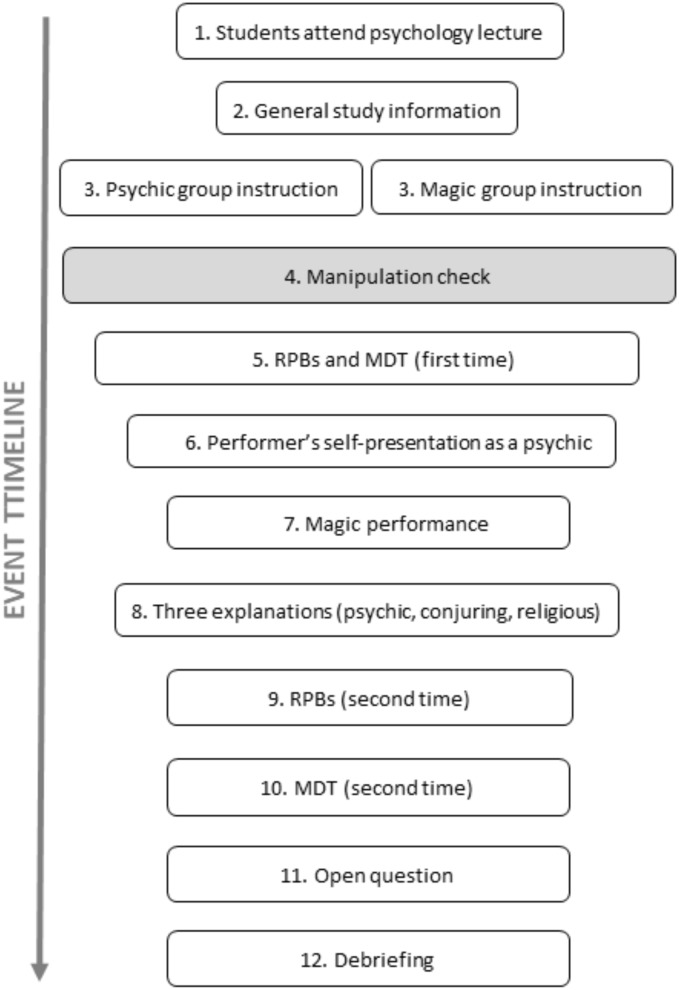
Sequence of events in Studies 1, 2, and 3. Note, the gray step (manipulation check) was only performed in Studies 2 and 3. Also, the results on the open question are not presented, because the formulation of the question (“Could you please describe what you saw in your own words?”) resulted in simple descriptions of the event as such. MDT, Mental Dice Task; RPBs, Revised Paranormal Belief Scale.

The sequence of events is depicted in Figure 1. After attending a psychology lecture, students were invited to take part in the experiment and were instructed to sit separately from each other by at least one empty seat. They were also asked not to communicate with each other in order to reduce the possibility of influencing one another. At this point, participants were all informed that they would experience a psychic performance (second event in Figure 1). The experimenter gave them the following verbal information:

“As you will be aware, the Anomalistic Psychology Unit at Goldsmiths has a keen interest in investigating psychic abilities. Over the years, we have carried out numerous experiments to test whether the claims made by psychics hold up on closer scrutiny. While most of the individuals tested so far generally fail these tests, we were very fortunate in that we did find one person who passed most of the preliminarily tests (8/10). His name is Jon and while not perfect, his performance was significantly better than chance (*p* < 0.0032). Jon has told us that he has been developing a presentation of his psychic abilities, and has asked us if he could present it to you and get your opinions and reactions. I thought that this would be very interesting, and so I agreed to let him do it.”

This introduction to the experiment, in particular the last sentence, was adapted from Benassi et al. (1980). Subsequent to these general instructions, participants received work booklets that contained all of the questionnaires and some additional information. Participants were randomly assigned to the magic or psychic condition (third event in Figure 1).

For the *magic condition*, the students received written information that the performance was carried out by a magician pretending to do a psychic performance. They read the following statement: “Jon is a professional magician who performs psychic performances for entertainment and has helped investigate fraudulent psychic phenomena. He is an active member of the Magic Circle, and has gained international respect for his conjuring ability. Jon has convinced many members of the APRU (Anomalistic Psychology Research Unit) with his conjuring skills. What you are about to see is a performance showing his conjuring deception skills and to the best of our knowledge does not involve any real psychic skills.”

For the *psychic condition*, the students received written information that the performance was carried out by a true psychic. They read the following statement: “Jon is a professional psychic who performs psychic performances for clients and has helped investigate psychic phenomena. He is an active member of the European Psychic Association, and has gained international respect for his psychic ability. Jon has convinced many members of the APRU with his psychic skills. What you are about to see is a performance showing his psychic skills and to the best of our knowledge does not involve any conjuring deception.”

After this written information, participants filled out the RPBs (Tobacyk, 2004; fifth event in Figure 1). Subsequently, they were asked to perform the MDT (Brugger et al., 1990; fifth event in Figure 1). Once completed, the performer introduced himself to the audience (sixth event in Figure 1). After the performance, the students were asked the three questions on how they would explain the event (eighth event in Figure 1). Afterward, participants completed a second time the RPBs (Tobacyk, 2004; ninth event in Figure 1), before doing the MDT again (Brugger et al., 1990; 10th event in Figure 1). Finally, they were asked the following open question: “Could you please describe what you saw in your own words?” (11th event in Figure 1), before being fully debriefed about the purpose of the experiment (12th event in Figure 1). Here, the performer explained the method behind the effect.

To summarize, the procedures were largely comparable across the three studies (see also Mohr et al., 2015), apart from two points. The first point was that we added a manipulation check (box in gray in Figure 1) for both Studies 2 and 3. This check consisted of asking participants two brief open questions after the framing text in order to ensure they had read and understood the information: (1) “Please summarize the instructions you have read in the space below” and (2) “Please write down what Jon’s profession is.” The second point was the change in the magic performance itself (divided into two stages; dice color guessing by “catching aura” of participant, and psychic reading, see under “Magic performance”).

### Data Analysis

Of the original 53 participants in Study 1, four participants were excluded. For one participant, demographic data (i.e., gender and age) were missing, and the other three were excluded because of their performance on the MDT – two did not complete the task (on neither occasion) and one entered the same number for each mental throw of the dice. Of the original 95 participants in Study 2, six participants were excluded because of incomplete MDT data: four did not complete the MDT (on neither occasion) and two participants did not complete the task on one of the occasions. Of the original 170 participants in Study 3, 47 participants were excluded. Forty-five were excluded because of incomplete MDT data: eight participants did not complete the sequence at least once; 37 participants missed at least two mental throws (range of missed throws = 2–59); and two participants were outliers, showing an over-proportional number of repetitions. In addition, one participant was excluded for having already completed the experiment before, and another participant for reporting “non-binary” gender. Since gender has an effect on our outcome measure (see also Aarnio and Lindeman, 2005; French and Wilson, 2007), the data for this participant were excluded.

Subsequent to this initial data cleaning, we examined the data for normality and outliers. The values for skewness and kurtosis were within the accepted range of ±2 for parametric analyses (Gravetter and Wallnau, 2014). No additional outliers were identified. Next, we performed Pearson correlations between age and our study variables (see Table 2), and *t*-tests to ascertain potential gender differences. To test our study hypotheses, we performed a series of repeated measures ANCOVAs with framing (psychic, magic) and study (Study 1–3) as between-subject measures, and testing session (before, after) as within-subjects’ variable on (i) PB scores (non-traditional belief, traditional belief); (ii) the number of repetitions in the MDT; and (iii) explanation scores (magic, psychic, religious). As will be seen in Section “Results”, we observed age and gender effects. Thus, we conducted all models with age and gender as covariates. We report Mauchly’s test in case the assumption of sphericity was violated. *Post hoc* comparisons were performed using false discovery rate (FDR, see Benjamini and Hochberg, 1995); corrected *p*-values are indicated as follow: *p*_corr_.

## Results

### Age and Gender Effects

The following section explores possible sex differences and age effects in the variables. There was no significant age difference [*t*(259) = -1.935, *p* = 0.054] between women (*M*_age_ = 20.52 years, *SD* = 5.04) and men (*M*_age_ = 22.07 years, *SD* = 6.57). Males as compared to females (i) generated more MDT repetitions before the performance, (ii) reported less traditional belief both before and after the performance, (iii) gave more conjuring explanations, and (iv) less psychic explanations (see Table 1). No gender differences were discerned for religious explanations, MDT repetitions after the performance, and non-traditional belief scores both before and after the performance.

**Table 1:** Means and standard deviations of the dependent measures for the total sample and the gender groups, separately.

		All		Women		Men		*t*-test	Effect
		(*N* = 261)		(*N* = 202)		(*N* = 59)			size
Measure		*M*	*SD*	*M*	*SD*	*M*	*SD*	Value	Cohen’s *d*
MDT repetitions	Before	5.42	4.28	5.05	4.15	6.66	4.54	-2.56*	-0.37
	After	5.63	4.89	5.37	4.68	6.53	5.49	-1.60	-0.23
TB	Before	3.95	2.02	4.22	2.05	3.04	1.59	4.66***	0.64
	After	3.88	2.10	4.18	2.12	2.87	1.65	4.99***	0.69
NTB	Before	2.69	1.01	2.73	0.98	2.56	1.11	1.15	0.16
	After	2.70	1.04	2.75	1.00	2.51	1.17	1.42	0.22
Explanations	Psychic	3.82	1.90	4.03	1.85	3.09	1.89	3.43***	0.50
	Conjuring	4.06	1.76	3.95	1.69	4.46	1.96	-1.82*	-0.28
	Religious	1.95	1.41	2.02	1.46	1.70	1.21	1.56	0.24


*MDT, Mental Dice Task; TB, traditional belief; NTB, non-traditional beliefs; before, before performance; after, after performance. T-tests and effect sizes refer to gender comparisons. ^∗^*p* < 0.05; ^∗∗∗^*p* < 0.001.*

With regard to age effects, Pearson correlations (Table 2) showed that older participants reported higher conjuring explanation scores and lower psychic explanation scores. Moreover, with increasing age, we found lower traditional belief scores, both before and after the performance.

**Table 2:** Pearson correlations between age, MDT repetitions, and self-report measures.

		Age	MDT repetitions		TB		NTB		Explanation		
			Before	After	Before	After	Before	After	Psychic	Magic	Religious
MDT repetitions	Before	0.071									
	After	0.037	0.716**								
TB	Before	-0.147*	-0.098	-0.079							
	After	-0.150*	-0.078	-0.061	0.973**						
NTB	Before	-0.083	-0.109	-0.154*	0.464**	0.506**					
	After	-0.089	-0.096	-0.116	0.422**	0.475**	0.924**				
Explanation	Psychic	-0.198**	-0.026	0.035	0.159*	0.197**	0.446**	0.553**			
	Magic	0.152*	0.029	0.028	0.015	-0.010	-0.169**	-0.236**	-0.511**		
	Religious	-0.113	-0.035	-0.027	0.392**	0.400**	0.394**	0.397**	0.355**	-0.221**	


*MDT, Mental Dice Task; TB, traditional belief; NTB, non-traditional beliefs. In cases of repeated assessments, the results are shown for before and after the performance. ^∗^*p* < 0.05 (two-tailed); ^∗∗^*p* < 0.01 level (two-tailed).*

### Relationships Between Dependent Measures

Self-report questionnaire scores as well as repetition avoidance scores correlated highly (all *r* values >0.70) when comparing the before and after measurements (Table 2). Also, higher non-traditional belief scores before the performance correlated with stronger repetition avoidance after the performance (Table 2). All correlations between the traditional belief and non-traditional belief scores were positive and significant (Table 2). Moreover, while the explanation scores were unrelated to performance in the MDT task, we note that higher traditional belief and non-traditional belief scores, both before and after the performance, were associated with higher psychic and religious explanation scores. Conjuring explanation scores, on the other hand, were unrelated to traditional belief scores (both before and after). With higher conjuring explanations, participants reported lower non-traditional belief scores both before and after the performance (Table 2). These latter results are also reflected in the inter-correlations between explanation scores, such that higher psychic explanation scores were associated with higher religious and lower conjuring explanation scores. Also, higher religious explanation scores were associated with lower conjuring explanation scores (Table 2).

### Effect of Study and Framing Group on Repetition Avoidance Before and After the Performance

The 2(framing) × 3(study) × 2(session) ANCOVA on the number of repetitions revealed a main effect of gender [*F*(1, 253) = 4.076, *p* = 0.045, $\eta_{p}^{2}$ = 0.016], with men showing less repetition avoidance than women (see Table 1), and a main effect of study (*F*(2, 253) = 3.065, *p* = 0.048, $\eta_{p}^{2}$ = 0.024). Pairwise comparisons showed more repetition avoidance in Study 3 (*M* = 4.86) than in both Study 1 (*M* = 6.35) [*M*_D_(se) = 1.488 (0.718), *p* = 0.039] and Study 2 (*M* = 6.02) [*M*_D_(se) = 1.157 (0.586), *p* = 0.049]. The difference between Study 1 and 2 was non-significant [*M*_D_(se) = 0.331 (0.758), *p* = 0.663]. The significant pairwise comparisons were, however, not significant after FDR correction (Study 3 vs. Study 1: *p*_corr_ = 0.074; Study 3 vs. Study 2; *p*_corr_ = 0.074). The model revealed no other significant main effects or interactions (all *F*-values <1.50).

### Effect of Study and Framing Group on Traditional Belief Scores Before and After the Performance

The 2(framing) × 3(study) × 2(session) ANCOVA on traditional belief scores showed a main effect for gender [*F*(1, 253) = 15.30, *p* < 0.001, $\eta_{p}^{2}$ = 0.057; women > men, see also Table 1], and a main effect of study [*F*(2, 253) = 3.217, *p* = 0.042, $\eta_{p}^{2}$ = 0.025]. Pairwise comparisons showed comparable traditional belief scores in Study 3 and Study 1 [*M*_D_(SE) = -0.769 (0.334), *p*_corr_ = 0.066], Study 3 and Study 2 [*M*_D_(SE) = -0.487 (0.273), *p*_corr_ = 0.114] as well as Study 1 and Study 2 [*M*_D_(SE) = -0.282 (0.353), *p*_corr_ = 0.425]. There were no other significant main effects or interactions (all *F*-values <3.30).

### Effect of Study and Framing Group on Non-traditional Beliefs Before and After the Performance

Since Mauchly’s test indicated that the assumption of Sphericity was violated [χ^2^_(df__=__2)_ = 75.58, *p* < 0.001], we report the Greenhouse–Geisser corrected estimates of Sphericity (𝜀 = 0.79). The 2(framing) × 3(study) × 2(session) ANCOVA on non-traditional beliefs revealed no significant main effects or interactions (all *F*-values < 2.60). Contrary to our *a priori* prediction, anticipating stronger performance in Study 2 and 3 as compared to Study 1, we found no main effect of study [*F*(2, 253) = 2.503, *p* = 0.084, $\eta_{p}^{2}$ = 0.019]. Pairwise comparisons showed that non-traditional belief scores were similar in Study 3 and Study 1 [*M*_D_(SE) = -0.379 (0.171), *p*_corr_ = 0.081], in Study 2 and Study 1 [*M*_D_(SE) = -0.310 (0.180), *p*_corr_ = 0.131], as well as in Study 2 and Study 3 [*M*_D_(SE) = -0.070 (0.139), *p*_corr_ = 0.617].

### Effect of Study and Framing Group on Explanation

The 2(framing) × 3(study) × 3(explanation) ANCOVA revealed a main effect for explanation [*F*(2, 402) = 8.706, *p* = 0.001, $\eta_{p}^{2}$ = 0.033] and study (*F*(2, 253) = 4.479, *p* = 0.012, $\eta_{p}^{2}$ = 0.034). These main effects interacted; explanation interacted with study [*F*(4, 402) = 8.446*, p* < 0.001, $\eta_{p}^{2}$ = 0.063], gender [*F*(2, 402) = 4.52, *p* = 0.018, $\eta_{p}^{2}$ = 0.018], and age [*F*(2, 402) = 4.53, *p* = 0.018, $\eta_{p}^{2}$ = 0.018]. There were no other significant main effects or interactions (all F-values < 2.98).

**FIGURE 2:**
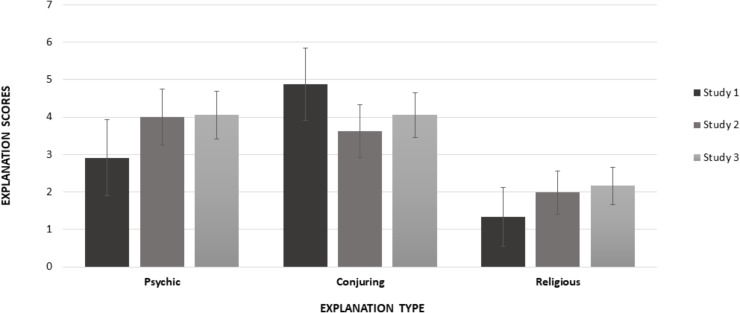
Mean explanation ratings by explanation type (psychic, conjuring, and religious) and study (1–3). Bars represent 95% confidence intervals of the mean.

We first unpacked the interaction between study and explanation (see Figure 2), with follow-up univariate ANCOVAs, controlling again for age and gender. The analysis of psychic explanation scores showed a significant main effect of age (*F*(1, 256) = 6.233, *p* = 0.013, $\eta_{p}^{2}$ = 0.024; see also Table 2), gender (*F*(1, 256) = 7.755, *p* = 0.006, $\eta_{p}^{2}$ = 0.029; see also Table 1), and study (*F*(2, 256) = 7.599, *p* = 0.001, $\eta_{p}^{2}$ = 0.056). For the main effect of study, pairwise comparisons showed lower psychic explanation scores in Study 1 compared to both Study 2 [*M*_D_(SE) = -1.084 (0.322), *p*_corr_ = 0.023)] and Study 3 [*M*_D_(SE) = -1.144 (0.306), *p*_corr_ < 0.001]. There was no difference between Study 2 and Study 3 [*M*_D_(SE) = -0.060 (0.249), *p*_corr_ = 0.810].

The ANCOVA of conjuring explanation scores showed a significant main effect of study (*F*(2, 256) = 8.374, *p* < 0.001, $\eta_{p}^{2}$ = 0.061). Pairwise comparisons showed higher conjuring explanation scores in Study 1 compared to both Study 2 [*M*_D_(SE) = 1.247 (0.305), *p*_corr_ < 0.001] and Study 3 [*M*_D_(SE) = 0.817 (0.289), *p*_corr_ = 0.009]. There was no difference between Study 2 and Study 3 [*M*_D_(SE) = -0.431 (0.235), *p*_corr_ = 0.088] (see Figure 2). All other main effects and interactions were not significant (all *F*-values <1.68).

Finally, the ANCOVA of religious explanation scores showed a significant main effect of study (*F*(2, 256) = 6.177, *p* = 0.002, $\eta_{p}^{2}$ = 0.046). Pairwise comparisons showed that religious explanation scores were lower in Study 1 than both Study 2 [*M*_D_(SE) = -0.644 (0.248), *p*_corr_ = 0.015] and Study 3 [*M*_D_(SE) = -0.825 (0.235), *p*_corr_ = 0.023] (Figure 2). Religious explanation scores did not differ between Study 2 and Study 3 [M_D_(SE) = -0.181 (0.193), *p*_corr_ = 0.393]. All other main effects and interactions were not significant (all *F*-values <1.60).

## Discussion

Numerous studies link PBs with cognitive biases (e.g., Fiske and Taylor, 2013; Irwin et al., 2013; Riekki et al., 2013). Yet, these studies have been predominately correlational in nature. Moving beyond these correlational designs, we investigated the potential of magic performances to test for causal relationships between PBs and PB-related cognitive biases (see also Benassi et al., 1980; Mohr et al., 2015). In the current study, we investigated whether people’s PBs and repetition avoidance changed when having a magic performance as central event (see also Mohr et al., 2018). In three subsequent studies, psychology students saw a magic performance in the classroom. About half of the sample was told that the performer was a psychic and the remainder that the performer was a conjuror. We assessed PBs and repetition avoidance before and after the performance. After the performance, participants rated to what extent they explain the performance in psychic, conjuring, and religious terms. Thus, the current design allowed us to elaborate on the paranormal potential of the magic performance, such as whether the performance, and/or its framing, would enhance explicit (RPBs scores) and implicit (repetition avoidance) correlates of PBs.

Our results showed neither framing nor timing (before–after) effects. This finding was consistent irrespective of (i) introducing a manipulation check (in which the participants summarized the framing text in their own words), (ii) providing more explicit definitions of “psychic” and “conjuring” in the instruction and framing texts, or (iii) increasing the strength of the paranormal nature of the magic performance.

One of our study goals was to explore which type of magic performance would result in an elevated amount of psychic explanations. A previous study showed that conventional magic tricks result in relatively low psychic explanations (Mohr et al., 2015). In the current series of studies, we added deceptive methods routinely used by magicians (Kuhn et al., 2014; Kuhn, 2019), including those giving the illusion of reading a person’s mind or communicating with the dead. The performance in Study 1 resembled the one in Mohr et al. (2015). The performer pretended to be able to “contact the dead” using his spiritual powers. The “contact” was made through the sudden lighting up of a candle (for more details, see Mohr et al., 2015). Students reported that the performance was not particularly strong in psychic terms. For Studies 2 and 3, the performer was in contact with a deceased person close to the confederate, and received accurate information from this deceased person. He did so after a general magic routine (knowing participants’ choices). In line with our goal, we could observe enhanced psychic potential of the magic performance, a significant increase in psychic explanations, as well as a drop in conjuring explanations in Studies 2 and 3 as compared to Study 1.

Despite the enhanced psychic potential of the magic performance in Studies 2 and 3, the lack of before–after differences on both explicit and implicit measures of PBs was disappointing. The possible reasons are, as yet, unclear to us (but see below some potential suggestions). The lack of framing effects (i.e., any difference between the psychic and conjurer group) was also unexpected, inconsistent with Benassi et al. (1980) and Mohr et al. (2015). It is possible that participants mixed up the different meanings of psychic and conjuring (there is evidence of this in participants’ responses). We changed the original framing text (Mohr et al., 2015) in the current studies to align the formulations of the psychic and conjuring framing texts (i.e., both framing texts had the same length, sentence structure, and message, and only differed in key statements/words). This alignment might have blurred the differences between the meaning of psychic and conjuring for some participants. We present a posteriori data that may support this explanation. Out of 261 participants, 96 rated the performance to be explained in both psychic *and* conjuring terms. The psychic and conjuring explanation scores were both above 4 on the seven-point Likert scale. For these participants, psychic and conjuring beliefs might be blurred or co-existent, much like it has been shown for the co-existence of psychic and scientific beliefs (Subbotsky, 2011; Legare et al., 2012). The latter explanation suggests the blurring or co-existence of conjuring and psychic reasoning. Intriguingly, Benassi et al. (1980) reported a similar finding: their participants’ explanations of the performance were inconsistent with the framing.

It is possible that some of our undergraduate students struggled to separate psychic from conjuring explanations, because of the nature of the event. The experimenters could observe that participants seemed not only highly attentive, but some seemed to react affectively (sad, empathic, angry). Any kind of framing information, particularly when comparably formulated, might become redundant when followed by an actual powerful event, such as a magic performance. This line of reasoning is corroborated by existing evidence. For instance, the more people endorsed PBs, the more they were likely to interpret a psychic performance to be a genuine example of a paranormal phenomenon (Hergovich, 2004). This relationship was not further influenced by previously provided information. Further studies show that initially provided information can become redundant (Olson et al., 2016; Thibault et al., 2018). In these studies, the researchers used a “placebo machine,” a mock MRI scanner. The participants were told that the scanner is not a real MRI scanner. Yet, the researchers mimicked a real MRI scan procedure informing participants that this procedure might impact psychological functioning. Importantly, the researchers noted that, a week later, many participants seemed to have already forgotten that the machine was fake (see also Olson, 2019).

We have some final noteworthy observations. First, while we did not replicate Mohr et al. (2015) finding that repetition avoidance was higher in the psychic compared to the conjuror group, we confirmed that higher non-traditional RPB scores correlated with stronger repetition avoidance (see also Brugger et al., 1990; Bressan, 2002). This correlation was significant only for the non-traditional RPBs scores before the performance and the repetition avoidance after the performance. Nevertheless, the correlation coefficients for the remaining correlations involving these measures were in the same direction (Table 2). Second, although we did not find, as in Mohr et al. (2015), that individuals undergoing psychic framing gave more psychic explanation and conjuring framing also gave more conjuring explanations, we did find that the RPBs scores and explanation scores correlated in meaningful ways: we replicated findings indicating that higher non-traditional RPBs scores correlated with higher traditional RPBs scores (Mohr et al., 2015; Prike et al., 2017). Also, psychic and religious explanation scores correlated with higher traditional RPBs scores and non-traditional RPBs scores, both before and after the performance (Mohr et al., 2015). We also found that lower conjuring explanation scores correlated with higher psychic and religious explanation scores. Finally, our mean RPBs scores and explanation scores were comparable to those in Mohr et al. (2015).

### Limitations and Challenges

Our findings need to be interpreted in light of several limitations and challenges. First, we refer to socio-demographic variables, such as variations in sample sizes, gender compositions, and age. Such variations are difficult to control when performing classroom studies. For a given study, we cannot determine who stays, nor what the gender and age composition will be. For instance, when looking at gender, we found that men as compared to women (i) produced more number repetitions (see also Spencer et al., 1999) prior to the magic performance, (ii) had fewer traditional beliefs (see also Aarnio and Lindeman, 2007; Mahlamäki, 2012), and (iii) provided more magic and less psychic explanations. For age, we found that older participants yielded both higher magic explanation scores and lower psychic explanation scores. Also, older participants within a student sample had lower traditional belief scores (see also Prike et al., 2017), both before and after the performance. Yet, our study was not designed to study age or gender effects. Therefore, we treated age and gender as covariates. Researchers can henceforth formulate *a priori* assumptions on age and gender, for laboratory studies in which age and gender ratios can be controlled.

Second, we refer to experimental variables. For instance, we used a seven-point Likert scale to measure PBs. Using an uneven scale might result in “I do not know” answers right in the middle of the scale. While we explicitly stated that “4” refers to being “uncertain,” we cannot be sure how this formulation was interpreted by participants. Using a Likert scale of an even number might be preferable. One could also use Visual Analog Scales to obtain a continuous variable (see, for example, Reips and Funke, 2008). Another concern could be that we used different magic performances and performers. Yet, keeping all conditions controlled is not possible when running live performances. Moreover, in the current series of studies, part of our aim was investigating different performances: “exploring magic performances that result in a significant amount of psychic explanations,” and by inference providing promising research material for before–after comparisons. Furthermore, the three studies were conducted within the course of a “science of magic” module (see also Mohr et al., 2015). Thus, participants knew that their lecturer (GK) studies magic, which might hamper spontaneous and honest responding. It is conceivable that participants made themselves appear more skeptical than they actually would be. Yet, none of our belief or explanation scores indicated a floor effect. One can only guess how much stronger the effects may be when taking away these contextual conditions (e.g., performing the study at an esoteric fair where people’s mind set is already primed to experience paranormal performances, see Van Elk, 2017).

We surmised in the discussion above that the performance was powerful enough to overcome framing effects. Thus, if we want framing to be processed such that it could influence explicit and implicit measures, we should consider when the framing text is presented and in which format. At the same time, there is no reason to assume that participants were so affectively or attentively captured to the point that it interfered with following instructions or tasks they were given. Participants completed the self-report questionnaires and answered questions after the performance coherently and reliably. Coherent answering can, for instance, be inferred from the high correlation coefficients between the before- and after-performance assessments and the replication of previous findings such as those on repetition avoidance (Brugger et al., 1990) and the correlations between non-traditional and traditional RPBs scores (Aarnio and Lindeman, 2007; Mohr et al., 2015; Prike et al., 2017).

### Conclusion

In the search for causal mechanisms of adult belief formation, we elaborated on an experimental design, with magic performance as its central event. We exposed undergraduate students to a magic performance in the classroom. We tested belief-related explicit (RPB scores) and implicit (repetition avoidance) measures before and after the performance and tested whether pre-existing beliefs impacted how this performance was explained in psychic, conjuring, and religious terms. We neither found before–after effects nor framing effects. Yet, we observed that the more a magic performance involved psychic routines, the more likely participants were to endorse psychic as well as religious explanations. In particular, we observed an increase in psychic explanations and a drop in conjuring explanations when performances involved such psychic routines. Thus, future studies should profit from a high probability of psychic explanations after such magic performances, and consider variables that lead to before–after effects as well as framing effects. Some directions have been proposed such as the co-existence of psychic and conjuring explanations in the same individual, and the potentially affective (see e.g., Lesaffre et al., 2018) and attention-capturing components of the magic performance.

## Data Availability

The raw data supporting the conclusions of this manuscript will be made available by the authors, without undue reservation, to any qualified researcher.

## Author Contributions

GK oversaw the data collection at Goldsmiths University. The experiments were designed by GK and CM (based on Benassi et al., 1980). AA-A, LL, and CM conducted the statistical analysis and interpretation of the results. LL was in charge of the writing of the full article, which included data treatment and interpretation. DR participated in data treatment and interpretation (including qualitative results to be reported elsewhere). All authors contributed to the critical and regular review of the present work. Moreover, all authors approved the submission of the current version.

## Conflict of Interest Statement

The authors declare that the research was conducted in the absence of any commercial or financial relationships that could be construed as a potential conflict of interest.The reviewer JO declared a past collaboration with the author GK.

## Footnotes

1 We work on the basis that the terms paranormal, supernatural, and magical beliefs can be used interchangeably (Lindeman and Svedholm, 2012).

2 When we refer to the terms magician, conjuror, or even psychic, we refer to the Robert Houdin magician acting style (see, for example, Lamont and Wiseman, 1999; Ortiz, 2006). In other words, we refer to someone who is playing as if he/she was a psychic (who, in fact, is not). This is radically different from someone who genuinely identifies him/herself as having paranormal (psychic) abilities.

3 For purposes of consistency in the current report, we re-label Benassi et al. (1980) “magic condition” “conjuror condition” or “conjuror group.”

4 Repetition avoidance was assessed [using a Mental Dice Task (MDT)], because it has been found to be higher in individuals with higher-PBs compared to individuals skeptical of PBs (Brugger et al., 1990). Thus, repetition avoidance represents a PB-related cognitive bias.

5 The Magic Circle promotes and advances the art of magic. It was founded in the United Kingdom in 1905. Its headquarter is in London. Membership is by merit. See also http://www.themagiccircle.co.uk.

6 The dice showed colors instead of numbers or dots on its sides.
