# Supplementary material for: Magic Performances – When Explained in Psychic Terms by University Students
Source: Front Psychol. 2018 Nov 6;9:2129. doi: 10.3389/fpsyg.2018.02129 (PMC6232384; doi:10.3389/fpsyg.2018.02129)
Supplement: Supplementary file 1 [file Table_1.DOCX]

Supplementary Material

Magic performances - When explained in psychic terms by university students

# Lise Lesaffre^*^, Gustav Kuhn, Ahmad Abu-Akel, Déborah Rochat, Christine Mohr

*** Correspondence:** Lesaffre Lise: lise.lesaffre@unil.ch

1. Framing instructions first published study (Mohr et al., 2015)

*Magic framing:* “Some magicians can perform exactly what psychics claim to be doing using ordinary stage trickery. In fact, Lee is not a real psychic, but a professional magician and member of the Magic Circle. What you are about to see is a demonstration of Lee’s conjuring skills.”

*Psychic framing:* “Lee has worked as a Psychic for several years. Lee is very highly regarded by the European Psychic Society and has astonished numerous well-known scientists by demonstrating his psychic abilities under tightly controlled conditions.”

1. Framing instructions of the 3 studies introduced in this paper

*Magic framing:* “Jon/Lee is a professional magician who performs psychic demonstrations for entertainment and has helped investigate fraudulent psychic phenomena. He is an active member of the Magic Circle, and has gained international respect for his conjuring ability. Jon has convinced many members of the APRU (Anomalistic Psychology Research Unit) with his conjuring skills. What you are about to see is a demonstration of his conjuring deception and to the best of our knowledge does not involve any real psychic skills.”

*Psychic framing:* “Jon/Lee is a professional psychic who performs psychic demonstrations for clients and has helped investigate psychic phenomena. He is an active member of the European Psychic Association, and has gained international respect or his psychic ability. Jon has convinced many members of the APRU with his psychic skills. What you are about to see is a demonstration of his psychic skills and to the best of our knowledge does not involve any conjuring deception.”

1. Event Interpretation Questionnaire (after performance)

**Please answer the questions bellow using the numbers indicated.**

**1 = Strongly Disagree, 2 = Moderately Disagree, 3 = Slightly Disagree, 4 = Uncertain, 5 = Slightly Agree,**

**6 = Moderately Agree, 7 = Strongly Agree.**

1. The performance was accomplished through paranormal, psychic or supernatural powers.

______

2. What you have seen has been accomplished by ordinary magic trickery.

______

1. What you have seen has been accomplished by a religious miracle.

______
